# Disease Germs

**Published:** 1872-08

**Authors:** I. N. Danforth

**Affiliations:** 74 South Morgan Street; Chicago


### Chicago Medical Journal.

A MONTHLY RECORD OF

*Medicine, Surgery and the Collateral Sciences.*

EDITED BY J. ADAMS ALLEN, M.D., LL.D.; AND WALTER HAY, M.D.

---

VOL. XXIX. — AUGUST, 1872. — NO. 8.

---

### Original Communications.

ARTICLE I. — *Disease Germs.* By I. N. DANFORTH, M.D., Chicago.

SECOND PAPER.

III. "Every dog must have his day," is a trite old saying, which is equally true of theories; and it may be added, without stretching the truth, that, in respect to longevity, dogs generally have the advantage of theories. Defunct theories, however, sometimes come to life again, but let us rejoice that defunct canines know no resurrection. The brevity of the sway of pathological theories, as well as the possibility of their subsequent revival, is well illustrated in the case of those first announced by Dr. J. H. Salisbury, of Cleveland.

In 1862, Dr. Salisbury was consulted by a gentleman who attributed an attack of measles to the inhalation of the dust from decaying wheat straw; and on the same day (Dec. 4th) this disease made its appearance in the military camp at Newark, Ohio, with which Dr. S. was officially connected. On examination, he found that the soldiers were sleeping upon straw beds, and, furthermore, that when the bedticks were filled, the straw was damp from recent exposure to snow. "Here were present all the conditions requisite for the formation of mould upon the straw, viz., organic decomposition, heat and moisture, and here were also visible the effects of the exposure of the men to such influences—nor could the disease be traced to any other source. The men came from different parts of the country, and had neither been exposed previous to enlistment, nor afterwards, to the contagious influence of the disease. * * * * "

With these observations before him, Dr. S. "deemed the subject one for further investigation, and accordingly procured the fungus growths of wheat, and the dust rising from them when agitated, for microscopical examination."* The microscope revealed "cells, spores, and sporangia, each element existing in greater or less quantity according to the amount of decomposition which the straw had undergone." He then took "clean white straw, free from fungi, packed it firmly in a small wooden box, wet it with a small amount of cold well water, and placed it with the lid firmly secured near the stove in his office, subjected to a temperature of from 60° to 75° Fah."

The cells and spores were promptly developed, and the doctor then "conceived the idea of inoculating himself," and did so with the effect of producing some catarrhal symptoms, and an eruption upon the face. Next his wife was inoculated, and substantially the same results followed. A boy six years of age who had been exposed to measles seventy-two hours before was then inoculated; this was followed by catarrhal symptoms, which subsided in a few days, and forty-two days afterwards no signs of measles had appeared. "This procedure was adopted in thirteen similar cases, with like results."

"I have not been able," says Dr. S., in conclusion, "to distinguish thus far any difference between the eruption and attendant symptoms of genuine measles and 'camp measles,' or straw measles."† Straw measles, however, generally make their appearance within from twenty-four to ninety-six hours after inhalation of the germs, while the genuine disease, as is well known, requires from eleven to fourteen days for its development. The observations and inferences of Dr. Salisbury concerning the germ origin of measles

* American Medical Times, vol. V., p. 134. † Op. cit., p. 135.

excited considerable *curiosity*, but nothing more than that, and so far as I am aware, no one now regards the straw fungus as capable of exciting an attack of genuine measles.

In the year 1866, Dr. Salisbury published a lengthy and very interesting article in the "American Journal of the Medical Sciences," on the cause of intermittent and remittent fevers. Dr. S. commenced his investigations by examining the expectoration of persons suffering from intermittent fever, and of those residing upon or frequenting malarial levels. He found "a great variety of zoosporoid cells, animalcular bodies, diatoms, dismidiæ, algoid cells and filaments, and fungoid spores. The only constant bodies, however, uniformly found in all cases, and usually in great abundance, were minute oblong cells, either single or aggregated, consisting of a distinct nucleus, surrounded by a smooth cell-wall with a highly clear, apparently empty space between the outside cell-wall and nucleus. Their peculiar appearance satisfied me (him) early that they were not fungoid, but cells of an algoid type, resembling *strongly those of the palmellæ*."*

Further observation and experiment demonstrated that the cells emanated from plants of a palmelloid type. After establishing this fact, the doctor undertook a careful series of investigations, in various localities, for the purpose of determining the relation of the palmellæ to paludal fevers. His observations and experiments are recorded with scrupulous care and exactness, and it would seem that they prove very clearly that the inhalation of the cells in question is pretty certain to bring on feverish symptoms, dryness of the fauces, and, if the exposure be sufficiently prolonged, true intermittent or remittent fever. Again, his observations demonstrated over again, what was already known, that persons residing in the wake of winds blowing over malarial soil, are almost certain to be attacked, while those located in the opposite direction are almost as likely to escape.

A detailed description of the different varieties of palmellæ is given in the work already cited (page 63), and on the same page, Dr. Salisbury remarks, "so far as I have examined (and my observations have been widely extended), I have never found a case of ague, *in situ*, where I did not find these plants growing near; and *vice versa*, I have never found these plants growing in any

* Am. Jour. Med. Sciences, January, 1866, p. 52.

locality but that (if such locality was inhabited) intermittent or remittent fever, or both, prevailed in proportion to their extent and profusion."

This theory of Dr. Salisbury, apparently so well founded and so carefully elaborated, attracted much attention and for a time found considerable favor with physicians. But it gradually lost its hold upon the profession, perhaps in some measure owing to the unfortunate fate of the straw-fungus theory, until it was either looked upon as the dream of an ingenious enthusiast, or forgotten altogether. Quite recently, however, a far distant observer has revived and given a new impetus to the doctrine of the germ-origin of miasm.

" M. Bolestra has reported his researches upon this subject, (the nature of miasm), to the French Academy of Sciences. The water of the Pontine Marshes, and of similar malarious regions, he found to contain, invariably, along with the common infusoria, a minute algoid vegetation, with an abundance of transparent, greenish-yellow spores $\frac{1}{1000}$ mm. in diameter. This vegetation develops slowly in pure water and at low temperatures, but rapidly in the heat of the sun and amid decomposing organic material. It floats upon the water, giving an iridescent film when young, and its spores are found in the air near the marshes, and even at Rome, being most abundant in warm weather and after a rain or during a fog, and least so in a cool, dry atmosphere. Dr. Bolestra regards these spores as the miasmatic agent in the production of the intermittent fevers for which the localities are badly celebrated."* This, it will be observed, is simply the Salisbury doctrine over again.

Count Castracane, the eminent Italian diatomist, has lately advanced a very ingenious theory as to the causation of miasmatic diseases, which, although not properly a germ theory, may be mentioned here. While endeavoring to ascertain what diatoms " live upon, and also why they suddenly die away nearly all at once," Count C. made the discovery that " nothing is so fatal to the life of marine or even brackish water diatoms as a sprinkling of pure fresh water. This he proved by repeated and carefully performed experiments. From this fact he comes to the very probable conclusion that the sudden dying away of myriads of diatoms,

* American Naturalist for June, 1872, p. 375.

besides, perhaps, myriads of other living creatures, during the rainy season (in Italy) might be, if not the only, at least, one of the most efficient causes of malaria." *

Certain observations relative to the spread of miasmatic diseases lend considerable support to the theory which attributes their origin to a germinal or material cause:

First, As already remarked, prevailing winds determine the direction in which they are propagated. They are *never* known to push their progress against the wind, but are always wafted in the direction of the air current.

Secondly, Belts of forest trees are very efficient agents in arresting their progress—their foliage apparently filtering the air and separating the germs—a result we should hardly expect did not the air contain some *material* source of infection.

Thirdly, Mountains or other high elevations of land have been observed to arrest the progress of paludal poisons.

Fourthly. Broad sheets of water, are, to some extent, protective, though, on the whole, less so than either forests or mountains. The protection afforded by water is in direct proportion to its extent—a narrow stream being vastly less efficient than a broad one.

Fifthly, It is a fact well known among oriental travelers—and especially by Englishmen residing or traveling in the British East Indian possessions, that sleeping under mosquito nets is very effective in preventing miasmatic diseases. Mr. E. L. Layard, a veteran traveler in the jungles, strongly advocates the constant use of mosquito nets, and pointedly deprecates neglecting this caution.† Other considerations equally potent might be added, but these are sufficient for present purposes.

Whether miasmatic diseases are caused by specific germs or not, is a question which must be investigated and settled mainly by the microscope. It should therefore be taken up and patiently worked out by microscopists having easy and frequent access to miasmatic regions. It will require a high magnifying power, an intimate acquaintance with low forms of vegetable and animal life, as well as months and possibly years of careful and pains-taking study. It will demand very many observations, made at

* Nature, vol. I, p. 481.

† Nature, vol. II, p. 143.

different seasons of the year, upon different soils, and in widely separated localities, which must be compared with each other, to convert the present probability into an established fact. But all this is well worth doing. If the germ-origin of paludal fevers be demonstrated, it will add one more *fact* to the few that are now struggling to keep each other cheerful in their loneliness, amid the strange mosaic of guess-work which we call Pathology.

74 South Morgan Street.

